# Central role of PI3K–SYK interaction in fibrinogen‐induced lamellipodia and filopodia formation in platelets

**DOI:** 10.1002/2211-5463.12149

**Published:** 2016-11-23

**Authors:** Raghvendra Singh

**Affiliations:** ^1^ Department of Chemical EngineeringIndian Institute of Technology KanpurIndia

**Keywords:** filopodia, lamellipodia, response time, Sos, WASP, WAVE

## Abstract

The WAVE complex‐1, a complex of WAVE, Abi1, NAP1, PIR121, HSPC300, RacGTP and Arp2/3 proteins, and WASP complex‐1, a complex of WASP, Cdc42, PIP2, and Arp2/3 proteins, are involved in lamellipodia and filopodia formation, respectively. It is known that the two complexes have opposite dynamics. Furthermore, Rac has two guanine nucleotide exchange factors, Vav and Sos, whose role in activating Rac is not well understood. In this work, by the construction of signaling network, analysis, and mathematical modeling, I show that Sos generates a pulse of WAVE complex‐1, decreasing the response time of WAVE complex‐1 formation upon the stimulation of platelets by fibrinogen. Furthermore, I also show that the dynamics of WAVE and WASP complexes depends on PI3K–SYK interaction. In the absence of this interaction, the WAVE complex‐1 does not form and the WASP complex‐1 remains at the initial, sustained level. Thus, I show the significance of the two protein/protein complexes: Sos and PI3K–SYK interaction, in fibrinogen‐induced lamellipodia and filopodia formation in platelets.

**Abbreviations**
WASP complex‐1: a complex of WASP, Cdc42, PIP2, and Arp2/3 proteins
WAVE complex‐1: a complex of WAVE, Abi1, NAP1, PIR121, HSPC300, RacGTP, and Arp2/3 proteins
WAVE complex‐2: a complex of Pir121, Nap, HSPC300, and Abi proteins

Platelets adhere to blood vessel walls during normal hemostasis or pathological occlusion of atherosclerotic arteries 1. Their activation and adhesion on the vascular surface are multistep processes involving cell surface molecules such as vWF receptor GPIb/V/IX, collagen receptor GPVI, and fibrinogen receptor α2bβ3 2. The deficiencies of fibrinogen and vWF cause bleeding disorders and, thus, these are the substrates of great physiological importance 1. Fibrinogen can cause both cell‐substrate adhesion and platelet–platelet aggregation by binding to integrin α2bβ3 1. While the vWF receptor's main function is to mediate high shear cell rolling on the surface, the primary function of the GPVI is to induce integrin α2bβ3or α2β1 inside‐out signaling through its FcRγ chain 2. This signaling causes the firm adhesion of platelets on to the injured wall, resulting in the formation of a cell monolayer 2. The adhesion is accomplished through the formation of adhesive protrusions, filopodia and lamellipodia. These are actin‐based structures involved in adhesion, cell migration, wound healing, chemotaxis, phagocytosis, cell signaling, and various other functions. The leading edges of a migrating or spreading cell, which contain actin protrusions that are parallel to substrate, are called lamellipodia. Lamellipodia have multiple important roles including adhesion to the substrate, macropinocytosis, and phagocytosis 3. On the other hand, filopodia are finger‐like protrusions from the cell surface and are composed of parallel bundles of actin polymer. The protrusions are extended as a result of the dynamics or threadmilling created by the barbed end actin polymerization and retrograde actin retraction. Arp2/3 complex has emerged as an important player that initiates actin polymerization in these structures. Besides Arp2/3, there are a number of signaling molecules, for example, WASP, WAVE, Rac, Cdc42, PIP2, PIP3, PI3K, etc. (Fig. 1A–E), that are involved in sensing and transmitting the requirement of these protrusions. Members of the rho family of GTPases, Rac and Cdc42, have been shown to regulate signal transduction pathways that link the formation of lamellipodium and filopodium to extracellular environment. Furthermore, these GTPases play a wider role in the response of the cell to various stimuli. For example, they regulate transcription factors through the activation of JNK and p38 pathways and affect stress and inflammation 4. Owing to a plethora of pathways these GTPases are involved, a large number of their guanine nucleotide exchange factors (GEF) and GTPase‐activating proteins (GAP) have been identified 4. Besides, the known GEFs and GAPs of Rac and Cdc42, phospholipids (PIP2, PIP3) and regulatory domain of PI3K also affect their nucleotide carrying state and their activity 5, 6, 7, 8, 9, 10. Another important molecule in fibrinogen/lamellipodia/filopodia signaling network is Spleen tyrosine kinase (SYK). SYK is abundantly found in hematopoietic cells and has been classically known to be involved with immunoreceptors in adaptive immune response 11. Later, many diverse roles of SYK, for example, in leukocyte function, integrin signaling, recognition of innate pathogens, fungi, bacteria and viruses, tissue damage, bone metabolism, platelet function, vascular development, allergy, and autoimmunity have been identified 11. Specifically, among these functions, SYK has been found to interact with β3 integrin and this interaction has been found to be important for SYK activation and lamellipodia formation in response to fibrinogen 12. More recently, SYK has also been identified as an important therapeutic target in rheumatoid arthritis 13, 14, type I diabetes 15, ischemia‐reperfusion injury 16, prostate cancer 17, acute myeloid leukemia 18, ovarian cancer 19 and chronic graft‐versus‐host disease 20. Interestingly, SYK has been found to have both pro‐ and anticancer roles 21, 22.

**Figure 1:**
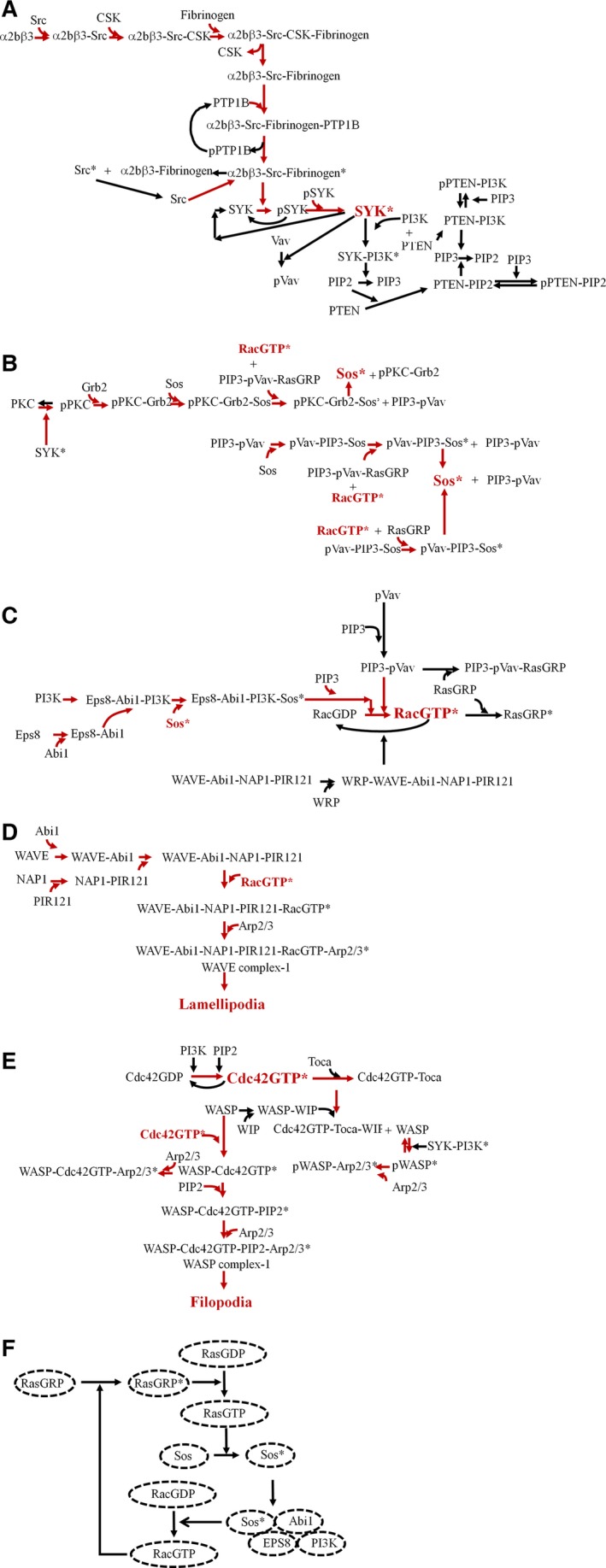
Fibrinogen induced WAVE and WASP complex formation network. The protein–protein interactions in fibrinogen/WAVE complex‐1/WASP complex‐1 network have been shown. The network has been divided into modules. *Represents active protein or active protein complex. (A) SYK activation module has been shown. The arrows in red color mark the pathway to SYK activation (B) Sos activation module has been shown. The arrows in red color mark the pathway to Sos activation (C) Rac activation module has been shown. The arrows in red color mark the pathway to Rac activation (D) Lamellipodia formation module has been shown. The arrows in red color mark the pathway to lamellipodia formation (E) Filopodia formation module has been shown. The arrows in red color mark the pathway to filopodia formation (F) Positive feedback loop between active Sos and active Rac through RasGRP and Ras.

It is known that there is coordination between the formation of the WAVE and WASP complexes in response to fibrinogen in platelets since both complexes are required as a part of a dynamic process accomplishing cell‐substrate adhesion and platelet–platelet aggregation. However, the protein or the protein–protein interaction critical for the above interplay is not yet known. Furthermore, there are two GEFs to Rac: Vav and Sos. Their roles in modulating the lamellipodia formation are also not yet known. To addresses these questions, I constructed the fibrinogen signaling network in platelets (Fig. 1A–E) and using mathematical modeling, predict the effect of various proteins and protein–protein interactions on the dynamics of the WAVE and WASP complexes. The model predicts that SYK phosphorylation (Fig. 1A), a key signaling event, and all other downstream steps occur after an initial delay. I further predict that Sos, a Ras GEF, is required for the rapid activation of the WAVE complex‐1, starting lamellipodia nucleation in response to fibrinogen. Moreover, the present analysis also predicts that the dynamics of the WAVE complex‐1 and the WASP complex‐1 formation is opposite of each other. In other words, while the WASP complex‐1 is present at a sustained level, the WAVE complex‐1 is absent in the absence of fibrinogen. In the presence of fibrinogen, while the WAVE complex‐1 increases to a sustained level, the WASP complex‐1 decreases to a very low level. Furthermore, I identify PI3K–SYK as the critical interaction modulating the dynamics.

## Materials and methods

### Construction of the signaling network

In resting platelets, Src is constitutively bound to integrin α2bβ3; however, it is inactive due to its phosphorylation at the inhibitory position Y529 by C‐terminal Src kinase, CSK 23. Upon fibrinogen binding to the receptor α2bβ3, CSK is released and phosphatase PTP‐1B binds to the receptor complex dephosphorylating Src at Y529 23, 24. Subsequently, Src gets autophosphorylated at the Y418 position in the activation loop and becomes active 23, 24. Following the Src activation, the active receptor complex phosphorylates SYK at tyrosine residues, leading to its activation 23, 25. When enzymatically active, SYK causes tyrosine phosphorylation of p110/p85 subunits of PI3K, increasing its catalytic activity 26, which converts PIP2 to PIP3. In contrast, the phosphatase PTEN through PI3K regulatory domain p85 27, 28 and PIP2 29 converts PIP3 back to the bisphosphate. Thus, PI3K plays mutually contrasting roles in maintaining the balance between the two phospholipids. Since actin polymerization happens near the membrane, this balance has an interesting role in filopodia and lamellipodia formation. In another upstream branch of α2bβ3 signaling network, SYK phosphorylates guanine nucleotide exchange factor Vav at tyrosine residues 30, 31, which is recruited by PIP3 at the membrane to convert RacGDP to RacGTP 5, 6. Furthermore, SYK tyrosine phosphorylates PKCα and PKCβ1, already autophosphorylated at serine residues, which makes accessible the binding site of PKCs for Sos bound Grb2 32. Similarly, active Vav through PIP3 binds to Sos 33. Furthermore, RacGTP converts RasGRP, a Ras guanine nucleotide exchange factor, to active form 34. RasGRP can also bind to Vav through PIP3 and is activated in a Rac‐dependent manner 33, 34. Since RasGRP increases the activity of Sos by the allosteric effect of RasGTP 35, I assume that the Sos bound to Vav and PKC is converted to an active form by RasGRP bound to Vav in the presence of the active Rac. Similarly, I assume that the active RasGRP converts Sos bound to Vav into an active form. Active Sos through complex with Eps8, Abi1, and PI3K converts RacGDP into RacGTP and the GEF activity of the complex is further increased by PIP3 7, 8. Thus, there are two Rac GEFs: first, the direct action of Vav and second, the Sos in complex with PI3K, Eps8, and Abi1. WAVE proteins have been shown to cause lamellipodia formation through the Arp2/3 complex in RacGTP‐dependent manner and WASP proteins, although dispensable 36, are involved in filopodia formation, which is dependent on Cdc42GTP 37. WAVE proteins (WAVE1 and WAVE2) are in a complex (WAVE complex‐2) with four other proteins: Pir121, Nap, HSPC300, and Abi 38, 39, 40, 41. This complex is autoinhibited and active Rac causes its activation. The WAVE2 complex‐2 also binds to WRP, which acts as a Rac GAP causing the termination of RacGTP‐mediated actin polymerization reaction 42.

PIP2 and a regulatory subunit of PI3K, p85, on the other hand, serve as alternatives to GEF toward Cdc42GDP, converting it to GTP form 9, 10. Cdc42GTP makes a complex with Toca‐1 while N‐WASP makes a complex with WIP which serves as an *in vivo* inhibitor of N‐WASP 43. The inhibitory effect of WIP is relieved by the interaction of the complex with Toca‐1 43. Although it is not very clear how this inhibition is relieved, I assume that the two complexes, Cdc42GTP‐Toca‐1 and N‐WASP‐WIP, interact with each other releasing N‐WASP. N‐WASP can be tyrosine phosphorylated 44, presumably by a kinase complex containing SYK 45, or it can bind to Cdc42GTP and/or PIP2 46. I postulate that it is the SYK‐PI3K complex that phosphorylates WASP. Phosphorylated N‐WASP, which is degraded 44, is active and so is the PIP2‐bound WASP. However, WASP alone is inactive and with Cdc42GTP is moderately active. PIP2 converts WASP‐Cdc42GTP to fully active form, which binds with the Arp2/3 complex. Thus, Arp2/3 nucleates actin polymers at the site of WASP‐Cdc42GTP‐PIP2 complex 43, 46, 47, 48, causing filopodia formation.

### Mathematical modeling

The signaling network causing WAVE complex‐1 and WASP complex‐1 formation in response to fibrinogen has been shown in Fig. 1A–E. As previously described 49, all phosphorylation/dephosphorylation reactions have been assumed to follow Michaelis–Menten kinetics. All phosphatases have been assumed to be in excess. It has been assumed that WASP is continuously produced with a rate α (nm·s^−1^) and degraded with a rate constant β (s^−1^) so that its steady‐state level, α/β, is maintained in the cell. Furthermore, I have assumed that phosphorylated WASP (pWASP) is degraded with a rate constant twice of that with which WASP is degraded. All rate constants have been assumed and are given in Text S1. There are 74 proteins and protein–protein complexes present in this signaling network. For each of them, I apply mass conservation as:

$$\frac{dC_{i}}{dt}=\sum_{j}r_{ij}$$

where, *C*_*i*_ is the intracellular concentration of a particular protein or protein complex *i* at time *t*, and *r*_*ij*_ is the rate of formation of the protein/protein complex *i* in the *j*th reaction. All reactions have been given in Text S1. The prestimulation protein levels have been assumed to be present in a wild‐type cell and have been given in Text S1. The resulting set of ODEs has been solved using the ode solver ODE113 of MATLAB R2014a (MathWorks, Natick, MA, USA). I have assumed that a cell is initially at a steady state. For all fibrinogen‐induced stimulation studies, I have taken the initial condition the same as the steady‐state condition in the absence of fibrinogen.

## Results

### The receptor complex causes an initial delay in start of the signaling

**Figure 2:**
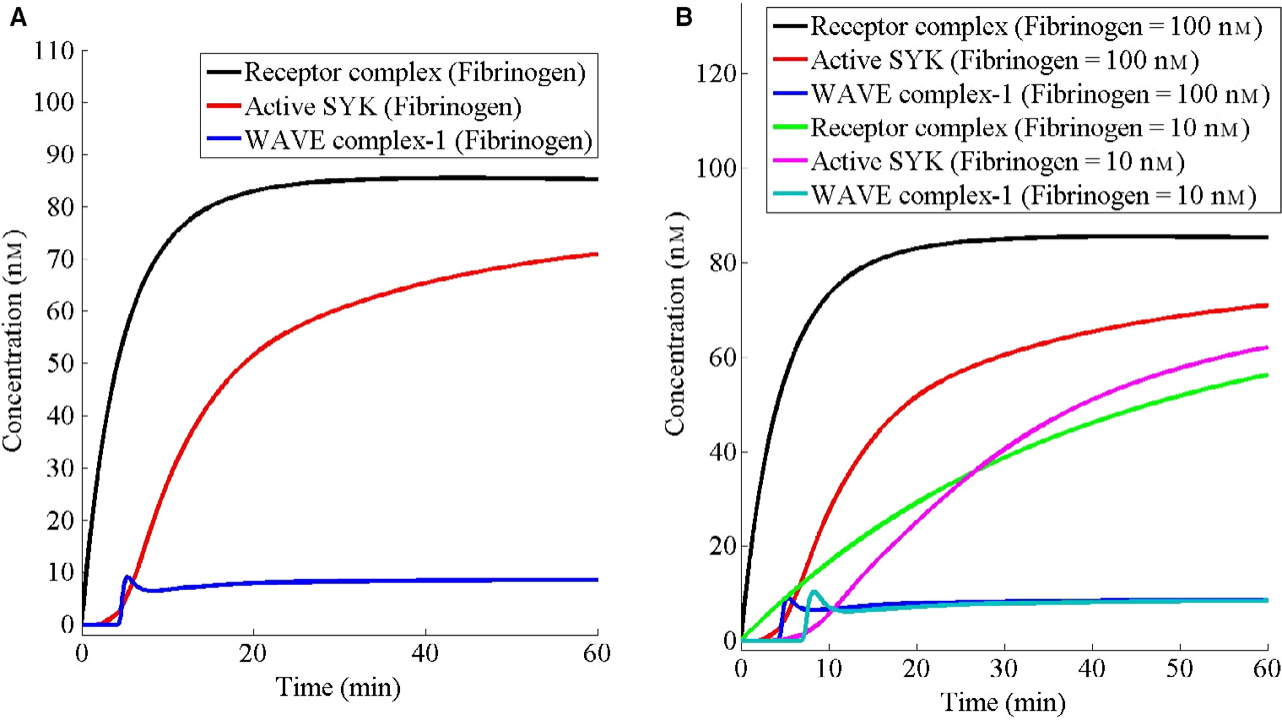
The receptor complex causes an initial delay in start of the signaling. Receptor complex consists of fibrinogen‐α2bβ3‐Src‐CSK. Initially, the cell has been assumed to be at the steady state in the absence of fibrinogen. (A) At *t* = 0, the cell has been stimulated by fibrinogen at a concentration of 100 nm (based on cell volume). (B) At *t* = 0, the cell has been stimulated with the indicated concentrations (based on the cell volume) of fibrinogen.

There are multiple reactions (Fig. 1A) at the fibrinogen receptor leading to SYK activation, a key phosphorylation event, which communicates the extracellular signal for the formation of lamellipodia and filopodia. First, I investigate the role of these reactions by examining the predicted time course of SYK activation and compare it with the time course of formation of fibrinogen‐α2bβ3‐Src‐CSK complex at the receptor. The dynamics shows that although the receptor complex forms without any delay, following a convex shape curve, activation of SYK starts with a delay, following a sigmoid curve (Fig. 2A,B), suggesting that the initial complex formation at the receptor may have an important cellular function. Interestingly, actin nucleation reaction in a pure *in vitro* reaction system has also been found to be sluggish 41, 47, 48.

### Fibrinogen modulates Rac, Cdc42 rho family GTPases, and Ras guanine nucleotide exchange factor, Sos

**Figure 3:**
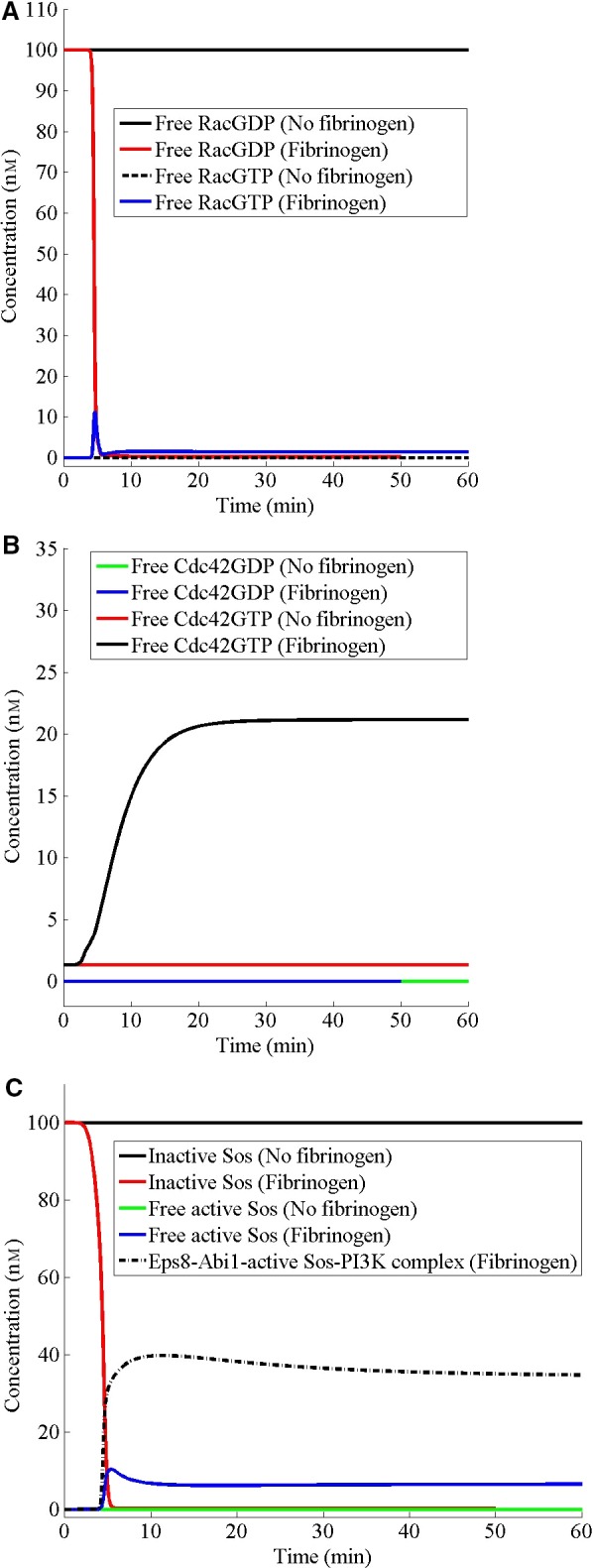
Fibrinogen modulates Rac, Cdc42 rho family GTPases and Ras guanine nucleotide exchange factor, Sos. (A–C) Initially, the cell has been assumed to be at the steady state in the absence of fibrinogen. For fibrinogen studies, at *t* = 0, the cell has been stimulated with 100 nm of fibrinogen (based on the cell volume). For ‘no fibrinogen’ studies, at *t* = 0, the cell has not been stimulated.

Since filopodia and lamellipodia extensions require actin polymerization, which involves rho family GTPases, Rac and Cdc42, I predict the dynamics of these proteins in the presence or absence of fibrinogen. Sos, which is a Ras family GEF, is not directly involved in actin polymerization but it, in a complex with Eps8, Abi1, and PI3K, works as a GEF to Rac. Thus, it indirectly modulates the actin polymerization through RacGTP. In the absence of fibrinogen, Rac is present only in the GDP form and the addition of fibrinogen rapidly decreases the RacGDP after an initial delay (Fig. 3A). However, it does not cause a corresponding increase in the free RacGTP. Instead, the RacGTP initially increases like a pulse, then settles down to a low level in the presence of fibrinogen (Fig. 3A). In contrast, Cdc42 is not present in the GDP form with or without fibrinogen (Fig. 3B). Interestingly, Cdc42GTP is present at a basal level in the absence of fibrinogen (Fig. 3B). Addition of fibrinogen causes an increase in Cdc42GTP to a sustained level (Fig. 3B) in contrast to the dynamics of RacGTP (Fig. 3A). Like Rac, Sos is present only in the inactive form in the absence of fibrinogen and fibrinogen causes a rapid decrease in inactive Sos concentration (Fig. 3C). However, following the addition of fibrinogen, more active Sos accumulates in complex with Eps8, Abi1, and PI3K than as free active Sos (Fig. 3C).

### Fibrinogen causes sustained activation of the lamellipodia‐forming complex and a concurrent decrease in the filopodia‐forming complex

**Figure 4:**
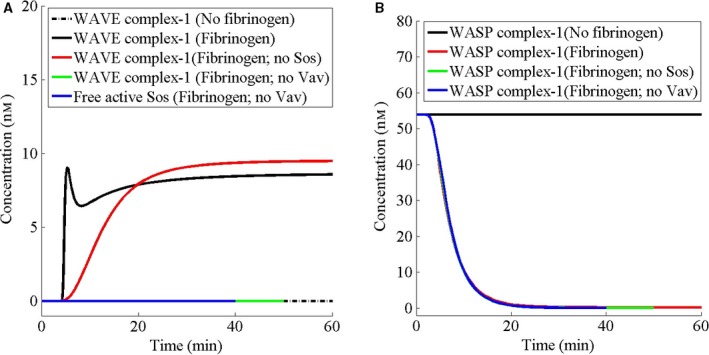
Fibrinogen causes sustained activation of the lamellipodia‐forming complex and a concurrent decrease in the filopodia‐forming complex. (A, B) Initially, the cell has been assumed to be at the steady state in the absence of fibrinogen. For fibrinogen studies, at *t* = 0, the cell has been stimulated with fibrinogen at a concentration of 100 nm (based on the cell volume). For ‘no fibrinogen’ studies, at *t* = 0, the cell has not been stimulated. For protein deletion studies, the amount of that protein has been set to 0 nm.

The WAVE, Abi1, NAP1, PIR121, HSPC300, RacGTP, and Arp2/3 complex (WAVE complex‐1) causes the nucleation of actin filaments for lamellipodia while WASP, cdc42GTP, PIP2, and Arp2/3 complex (WASP complex‐1) causes nucleation of the actin filament for filopodia. The dynamics of these complexes in the presence or absence of fibrinogen was studied. Furthermore, the role of two GEFs, Vav and Sos, in the predicted dynamics of the WAVE and WASP complexes was investigated. In the absence of fibrinogen, the WAVE complex‐1 is not present while the WASP complex‐1 is present at a fixed level (Fig. 4A,B). Following the addition of fibrinogen, WAVE complex‐1 increases rapidly after the initial delay, reaching a sustained level while the WASP complex‐1 decreases to a low level (Fig. 4A,B). The dynamics of the WAVE complex‐1 consists of a rapid pulse followed by a slow increase phase, reaching a plateau. Interestingly, in the absence of Sos, the second GEF to Rac, the dynamics of WAVE complex lacks the rapid pulse and is sluggish, suggesting a role of Sos in generating the pulse of the WAVE complex‐1 and modulating the response time of the complex formation. On the other hand, in the absence of Vav, the first GEF to Rac, the WAVE complex does not form at all, underlying a major role of Vav in lamellipodia formation. Interestingly, in the absence of Vav, active Sos also does not form (Fig. 4A), suggesting a role of Vav in Sos activation and further supporting the role of Sos in the WAVE complex‐1 pulse generation. In contrast, neither Sos nor Vav has any significant effect on the dynamics of the WASP complex‐1, since these proteins are involved in lamellipodia formation but not in filopodia formation (Figs 4A,B, and 1C,D vs. 1E).

### Decreasing fibrinogen concentration delays the lamellipodia nucleation

**Figure 5:**
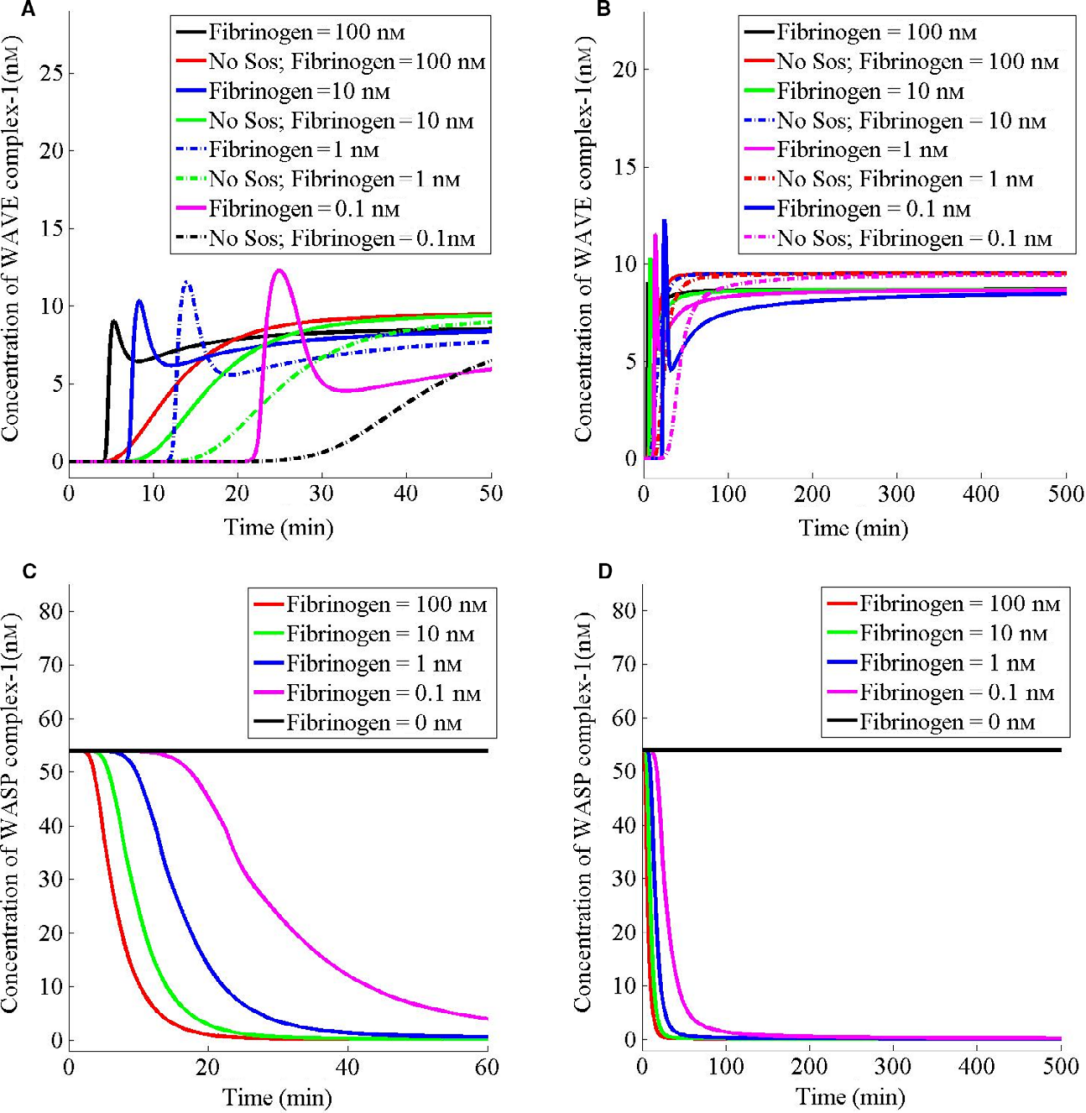
Decreasing fibrinogen concentration delays the lamellipodia nucleation. (A–D) Initially, the cell has been assumed to be at the steady state in the absence of fibrinogen. At *t* = 0, the cell has been stimulated with the indicated concentrations (based on the cell volume) of fibrinogen. For protein deletion studies, the amount of that protein has been set to 0 nm.

Next, I varied the fibrinogen concentration and examined the dynamics of lamellipodia‐ and filopodia‐nucleating complexes. I found that decreasing fibrinogen delays the formation of the WAVE complex‐1 both in the presence and absence of Sos (Fig. 5A). Furthermore, decreasing fibrinogen concentration also delays the fibrinogen‐induced decrease in the WASP complex‐1 (Fig. 5C). The steady state of the WAVE complex‐1 is different in the presence and absence of Sos (Fig. 5B). However, the WAVE complex‐1 achieves the same steady state for all concentrations of fibrinogen, which were tested in 100–0.1 nm range (Fig. 5B), suggesting that in this range, the lamellipodia nucleation is robust. Similarly, the WASP complex‐1 achieves the same steady state for all concentrations of fibrinogen, which were tested in 100–0.1 nm range (Fig. 5D).

### PI3K–SYK interaction plays a central role in filopodia and lamellipodia nucleation

**Figure 6:**
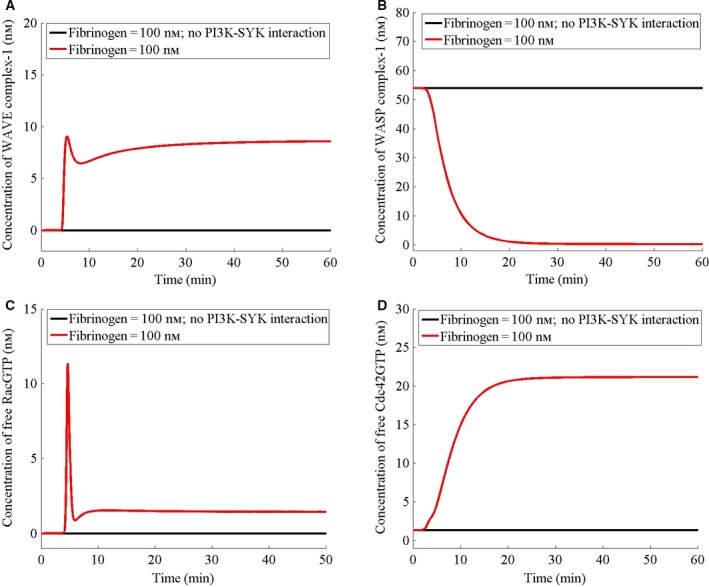
PI3K–SYK interaction plays a central role in filopodia and lamellipodia nucleation. (A–D) Initially, the cell has been assumed to be at the steady state in the absence of fibrinogen. For fibrinogen studies, at *t* = 0, the cell has been stimulated with fibrinogen at a concentration of 100 nm (based on the cell volume). For ‘no PI3K–SYK interaction’ studies, the active SYK and PI3K binding reaction has been abolished.

Next, I examine the protein–protein interactions responsible for the fibrinogen‐induced dynamics of the WAVE and WASP complexes and find that abolishing the interaction between PI3K and SYK abolishes the fibrinogen‐induced decrease in the WASP complex‐1 (Fig. 6B). Furthermore, abolishing PI3K–SYK interaction nullifies the fibrinogen‐induced increase in the WAVE complex‐1 (Fig. 6A) and the free RacGTP (Fig. 6C). It also abolishes fibrinogen‐induced dynamics of the free Cdc42GTP although Cdc42GTP remains at the basal level (Fig. 6D). Thus, in the absence of PI3K and SYK interaction, the fibrinogen does not cause lamellipodia nucleation neither does it modulate the filopodia nucleation, signifying a central role of PI3K–SYK in fibrinogen‐platelet signaling.

## Discussion

The model predicts that there is a delay in SYK activation with respect to the time of formation of fibrinogen, integrin α2bβ3, Src, and CSK complex at the receptor. At the receptor, multiple reactions (Fig. 1A) upstream of SYK activation take place. Besides being part of the signal regulatory mechanism, these reactions may serve to create a time lag before the start of actin threadmilling process, which is an energy intensive polymerization–depolymerization reaction. Toward the experimental observation of the delay in SYK activation, Tohyama *et al*. 50 studied its tyrosine phosphorylation in megakaryoblastic leukemia cells, a platelet cell line, on both solid phase and soluble fibrinogen. They found that while soluble fibrinogen triggers SYK phosphorylation within 1 min, solid phase fibrinogen causes its significant phosphorylation as late as 30 min after the adhesion 50, consistent with our prediction.

Ras GTPase is activated in several growth factor signaling, which activates Erk1/2 51, 52. The growth factors have also been shown to activate JNK through Rac 53. The crosstalk between Erk1/2 and JNK signaling has been orchestrated by Sos 54, a guanine nucleotide exchange factor to Ras, which has also been identified as a GEF to Rac when in a complex with three other proteins Eps8, Abi1, and PI3K 7, 8. On the other hand, Vav is a direct guanine nucleotide exchange factor to Rac 5, 6. The role of these two guanine nucleotide exchange factors of Rac in fibrinogen/platelet signaling is not well understood. Here, I predict that Sos through Rac is responsible for the generation of a rapid pulse of WAVE complex‐1, which nucleates actin lamellipodia. In the absence of Sos, WAVE complex‐1 formation is devoid of the pulse and sluggish. Sos has two binding sites 55 for Ras: (a) the allosteric site and (b) the catalytic site. By binding to the allosteric site, Ras increases the activity of Sos and at the catalytic site Sos activates Ras, creating a positive feedback loop between Ras and Sos. RasGRP is another GEF to Ras. RasGRP is activated by Rac 34. At the catalytic site of Sos either Ras or Rac can bind 56. However, Ras can bind to the allosteric sites of Sos while Rac is bound to the catalytic site of Sos. Thus, there is another positive feedback loop between the active Sos and the active Rac involving RasGRP and Ras (Fig. 1F). By this positive feedback loop, active Sos and active Rac may mutually increase each other's concentration, generating the rapid pulse of the WAVE complex‐1. Thus, there is an initial delay in signaling but once a decision of actin polymerization has been made by the cell, a rapid pulse of WAVE complex‐1 is generated through Sos. Innocenti *et al*. 56 show a dual role of Sos in activating Ras and Rac. In response to growth factors, while Ras is transiently activated by Sos, like a pulse, Rac activation is sustained 56. This is similar to our prediction that Sos generates a pulse while Vav generates a sustained activation of the WAVE complex‐1. In the absence of Vav, we should see a transient pulse of the WAVE complex‐1, which is due to Sos. However in the absence of Vav, the active Sos is also absent (Fig. 4A). Therefore, in the absence of Vav, the WAVE complex‐1 pulse is null.

Nucleation of a new actin filament is less favorable than the addition of actin monomers at the barbed and the pointed ends of the filament 57. Between the two ends, monomer addition at the barbed end is both faster and more favorable 57. Furthermore, the critical concentration required for monomer addition at the barbed end is lower than that at the pointed end. Due to this difference and sequestration of the monomer by profilin and thymosin‐β4, the concentration of the free actin monomer is maintained well below the critical concentration at the pointed end and higher than the critical concentration at the barbed end, where profilin‐actin can be incorporated in the elongation, leading to a net addition at the barbed end and a net dissociation at the pointed end, which results in a slow threadmilling from the barbed toward the pointed end 57. Owing to the high concentration of monomeric actin and the possibility of continuous polymerization, barb ends are capped by capping protein and gelsolin and the process of uncapping and nucleation is regulated through signaling. Arp2/3 complex is responsible for the nucleation but it has low intrinsic activity and is activated by binding of adapter proteins WASP and WAVE. Like Arp2/3, WASP and WAVE are autoinhibited and activated through signaling involving Cdc42, and Rac, respectively. Although both Rac and Cdc42 are activated by the same signaling network, it is found that the inhibition of lamellipodia formation, which occurs upon the deletion of the capping protein, may result in explosive formation of filopodia 58. Thus, there is an intrinsic dynamic between the two forms of the cytoskeleton. Similarly, I predict that in the absence of fibrinogen, there is a steady presence of a filopodia‐nucleating complex (WASP complex‐1) and absence of a lamellipodia‐nucleating complex (WAVE complex‐1). The addition of fibrinogen causes an increase in the WAVE complex‐1 to a sustained level and a concurrent decrease in the WASP complex‐1 to a very low level (Fig. 4A,B), exhibiting the opposite dynamics. Furthermore, I predict that fibrinogen causes a concurrent increase in free Cdc42GTP (Fig. 3B). However, the effect of fibrinogen on the WASP complex‐1 and Cdc42GTP dynamics is abolished in the absence of PI3K‐SYK interaction (Fig. 6B,D), suggesting that the dynamics of WASP complex‐1 in the presence of fibrinogen is due to the effect of the PI3K–SYK interaction on Cdc42GTP. Similar to filopodia formation, phagocytosis by macrophages requires activation of Cdc42 at the advancing edge of the phagocytic cup and deactivation of Cdc42 at the base of the cup 59. Beemiller *et al*. 59 found that inhibition of PI3K causes persistent activation of Cdc42 and stalled phagocytic cup, which is in agreement with our prediction that in the absence of the SYK–PI3K interaction, the WASP complex‐1 becomes persistently active (Fig. 6B). Similarly, I predict that the effect of fibrinogen on WAVE complex‐1 formation is abolished in the absence of SYK–PI3K interaction and the WAVE complex‐1 does not form in the absence of this interaction, which is in agreement with Kato *et al*. 60 and Weering *et al*. 61, who show that inhibiting PI3K inhibits lamellipodia extension. Furthermore, Woodside *et al*. 12 have shown that SYK interacts with the cytoplasmic tail of β3 integrin and disruption of this physical association abolishes SYK activation and lamellipodia formation in response to fibrinogen, consistent with our prediction. Although the dynamics of the two complexes in general turn out to be opposite of each other in response to fibrinogen, their formation does not share all the molecules. For example, Vav and Sos regulate only the WAVE complex‐1 formation in response to fibrinogen. Furthermore, the sum of WAVE and WASP complexes is not a constant (data not shown). Thus, the opposite dynamics of the two complexes may be an intrinsic property of the signaling network.

In summary, I predict that the PI3K–SYK interaction is central to fibrinogen‐induced dynamics of the WAVE and the WASP complexes since in the absence of PI3K–SYK interaction, the interplay between the two complexes is abolished and fibrinogen has no effect on lamellipodia and filopodia nucleation. Furthermore, I predict that Sos generates a pulse of WAVE complex‐1 in response to fibrinogen, reducing the response time of lamellipodia formation. My predictions agree with the experimental findings, although the predicted role of Sos remains to be verified experimentally.

## Supporting information


**Text S1.** Reactions, rate of reactions, rate constants, and initial concentrations.
