# Supplementary material for: Central role of PI3K–SYK interaction in fibrinogen‐induced lamellipodia and filopodia formation in platelets
Source: FEBS Open Bio. 2016 Nov 23;6(12):1285–96. doi: 10.1002/2211-5463.12149 (PMC5324771; doi:10.1002/2211-5463.12149)
Supplement: Supplementary file 1 — Text S1. Reactions, rate of reactions, rate constants, and initial concentrations. [file FEB4-6-1285-s001.doc]

**Text S1**

**Reactions in the fibrinogen induced WAVE/WASP complex signaling network**

$$R+\text{ Src }\Leftrightarrow\text{ RS; (1)}$$

$$\text{R}S+\text{ CSK }\Leftrightarrow\text{ RSC; (2)}$$

$$\text{F}+RSC\text{ }\Leftrightarrow\text{ FRSC; (3)}$$

$$\text{F}RSC\text{ }\Leftrightarrow\text{ FRS }+\text{ CSK; (4)}$$

$$\text{FRS}+PTP\Leftrightarrow\text{ FRSP; (5)}$$

$$\text{F}RSP\text{ }\Leftrightarrow\text{ FRSa}+\text{ pP; (6)}$$

$$F\text{RSa }\Leftrightarrow\text{ FR }+\text{ aSrc; (7)}$$

$$\text{FR}+Src\text{ }\Leftrightarrow\text{ FRSa; (8)}$$

$$pP\xrightarrow{}\text{PTP; (9)}$$

$$\text{ }a\text{Src}\xrightarrow{}\text{Src; (10)}$$

$$\text{ }p\text{SYK}\xrightarrow{}\text{SYK; (12)}$$

$$\text{ }a\text{SYK}\xrightarrow{}\text{SYK; (14)}$$

$$aSYK+\text{ PI3K }\Leftrightarrow\text{ aSYK\_PI3K; (15)}$$

$$PTEN+\text{ PI3K }\Leftrightarrow\text{ PTEN\_PI3K; (17)}$$

$$PTEN+\text{ PIP2 }\Leftrightarrow\text{ PTEN\_PIP2; (18)}$$

$$\text{pVav }\xrightarrow{}\text{Vav; (21)}$$

$$PIP3+\text{ pVav }\Leftrightarrow\text{ PIP3\_pVav; (22)}$$

$$\text{pPKC }\xrightarrow{}\text{PKC; (25)}$$

$$pPKC+\text{ Grb2 }\Leftrightarrow\text{ pPKC\_Grb2; (26)}$$

$$pPKC\_Grb2+\text{ Sos }\Leftrightarrow\text{ pPKC\_Grb2\_Sos; (27)}$$

$$PIP3\_pVav+\text{ Sos }\Leftrightarrow\text{ pVav\_PIP3\_Sos; (28)}$$

$$RasGrp+\text{ racGTP }\Leftrightarrow\text{ aRasGrp; (29)}$$

$$PIP3\_pVav+\text{R}asGrp\text{ }\Leftrightarrow\text{ PIP3\_pVav\_RasGrp; (30)}$$

$$pPKC\_Grb2\_Sos+\text{ PIP3\_pVav\_RasGrp}+\text{ racGTP }\Leftrightarrow\text{ pPKC\_Grb2\_Sosa }+\text{ PIP3\_pVav; (31)}$$

$$pVav\_PIP3\_Sos+\text{ PIP3\_pVav\_RasGrp}+\text{ racGTP }\Leftrightarrow\text{ pVav\_PIP3\_Sosa }+\text{ PIP3\_pVav; (32)}$$

$$pVav\_PIP3\_Sos+\text{ }a\text{RasGrp }\Leftrightarrow\text{ }p\text{Vav\_PIP3\_Sosa; (33)}$$

$$pPKC\_Grb2\_Sosa\Leftrightarrow\text{ pPKC\_Grb2 }+\text{ Sosa; (34)}$$

$$pVav\_PIP3\_Sosa\text{ }\Leftrightarrow\text{ pVav\_PIP3 }+\text{ Sosa; (35)}$$

$$N\text{AP1 }+\text{ PIR121 }\Leftrightarrow\text{ }N\text{AP1\_PIR121; (36)}$$

$$W\text{AVE }+\text{ Abi1 }\Leftrightarrow\text{ }W\text{AVE\_Abi1; (37)}$$

$$W\text{AVE\_Abi1 }+\text{ NAP1\_PIR121 }\Leftrightarrow\text{ }W\text{AVE\_Abi1\_NAP1\_PIR121; (38)}$$

$$W\text{AVE\_Abi1\_NAP1\_PIR121}+\text{ WRP }\Leftrightarrow\text{ WRP\_}W\text{AVE\_Abi1\_NAP1\_PIR121; (39)}$$

$$E\text{ps8 }+\text{ Abi1 }\Leftrightarrow\text{ }E\text{ps8\_Abi1; (41)}$$

$$E\text{ps8\_Abi1 }+\text{ PI3K }\Leftrightarrow\text{ }E\text{ps8\_Abi1\_PI3K; (42)}$$

$$E\text{ps8\_Abi1\_PI3K }+\text{ Sosa }\Leftrightarrow\text{ }E\text{ps8\_Abi1\_Sos\_PI3K; (43)}$$

$$W\text{AVE\_Abi1\_NAP1\_PIR121}+\text{ racGTP }\Leftrightarrow\text{ }W\text{AVE\_Abi1\_NAP1\_PIR121\_racGTP; (46)}$$

$$W\text{AVE\_Abi1\_NAP1\_PIR121\_racGTP}+\text{ Arp2/3 }\Leftrightarrow\text{ }W\text{AVE\_Abi1\_NAP1\_PIR121\_racGTP\_Arp; (47)}$$

$$W\text{ASP }+WIP\text{ }\Leftrightarrow\text{ }W\text{ASP\_WIP; (50)}$$

$$c\text{dc42GTP }+\text{ Toca }\Leftrightarrow\text{ }cdc42GTP\_Toca\text{; (51)}$$

$$cdc42GTP\_Toca+W\text{ASP\_}WIP\text{ }\Leftrightarrow\text{ }c\text{dc42GTP\_Toca\_WIP }+\text{ WASP; (52)}$$

$$WASP+\text{ cdc42GTP }\Leftrightarrow\text{ WASP\_cdc42GTP; (54)}$$

$$WASP\_cdc42GTP+\text{ PIP2 }\Leftrightarrow\text{ WASP\_cdc42GTP\_PIP2; (55)}$$

$$WASP\_cdc42GTP\_PIP2+\text{ Arp2/3 }\Leftrightarrow\text{ WASP\_cdc42GTP\_PIP2\_Arp; (56)}$$

$$\text{pPTEN\_PI3K }\xrightarrow{}\text{PTEN\_PI3K; (58)}$$

$$\text{pPTEN\_PIP2 }\xrightarrow{}\text{PTEN\_PIP2; (60)}$$

$$\text{pWASP }\xrightarrow{}\text{WASP; (61)}$$

$$\text{NULL }\xrightarrow{}\text{WASP; (62)}$$

$$WASP\_cdc42GTP+\text{ Arp2/3 }\Leftrightarrow\text{ WASP\_cdc42GTP\_Arp; (63)}$$

$$pWASP+\text{ Arp2/3 }\Leftrightarrow\text{ pWASP\_Arp; (64)}$$

**Rate of reactions**

$$\text{ r}_{1}=\text{ k}_{1}\text{ R. Src - k}_{\text{-1}}\text{ RS }$$

$$\text{ r}_{2}=\text{ k}_{2}\text{ RS. CSK - k}_{\text{-2}}\text{.RSC }$$

$$\text{ r}_{3}=\text{ k}_{3}\text{ F. RSC - k}_{\text{-3}}\text{FRSC }$$

$$\text{ r}_{4}=\text{ k}_{4}\text{ FRSC - k}_{\text{-4}}\text{.FRS. CSK }$$

$$\text{ r}_{5}=\text{ k}_{5}\text{ FRS. PTP - k}_{\text{-5}}\text{.FRSP }$$

$$\text{ r}_{6}=\text{ k}_{6}\text{ FRSP - k}_{\text{-6}}\text{.FRSa. pP }$$

$$\text{ r}_{7}=\text{ k}_{7}\text{ FRSa - k}_{\text{-7}}\text{.FR. aSrc }$$

$$\text{ r}_{8}=\text{ k}_{8}\text{ FR. Src - k}_{\text{-8}}\text{.FRSa }$$

$$\text{r}_{9}=\text{ }\frac{\text{v}_{9}.\begin{matrix} \end{matrix}pP\begin{matrix} \end{matrix}}{\text{k}_{9}+pP}\text{ }$$

$$\text{r}_{10}=\text{ }\frac{\text{v}_{10}.aSrc}{\text{k}_{10}+aSrc}\text{ }$$

$$\text{r}_{11}=\text{ }\frac{\text{v}_{11}.SYK.FRSa}{\text{k}_{11}+SYK}\text{ }$$

$$\text{r}_{12}=\text{ }\frac{\text{v}_{12}.pSYK}{\text{k}_{12}+pSYK}\text{ }$$

$$\text{r}_{13}=\text{ }\frac{\text{v}_{13}.pSYK.pSYK}{\text{k}_{13}+pSYK}\text{ }$$

$$\text{r}_{14}=\text{ }\frac{\text{v}_{14}.aSYK}{\text{k}_{14}+aSYK}\text{ }$$

$$\text{ r}_{15}=\text{ k}_{15}\text{ aSYK. PI3K - k}_{\text{-15}}\text{.aSYK\_PI3K }$$

$$\text{r}_{16}=\text{ }\frac{\text{v}_{16}.aSYK\_PI3K.PIP2}{\text{k}_{16}+PIP2}\text{ }$$

$$\text{ r}_{17}=\text{ k}_{17}\text{ PTEN. PI3K - k}_{\text{-17}}\text{PTEN\_PI3K }$$

$$\text{ r}_{18}=\text{ k}_{18}\text{ PTEN. PIP2 - k}_{\text{-18}}\text{PTEN\_PIP2 }$$

$$\text{r}_{19}=\text{ }\frac{\text{v}_{19}.(PTEN\_PI3K+PTEN.PIP2).PIP3}{\text{k}_{19}+PIP3}\text{ }$$

$$\text{r}_{20}=\text{ }\frac{\text{v}_{20}.VAV1.aSYK}{\text{k}_{20}+VAV1}\text{ }$$

$$\text{r}_{21}=\text{ }\frac{\text{v}_{21}.pVAV1}{\text{k}_{21}+pVAV1}\text{ }$$

$$\text{ r}_{22}=\text{ k}_{22}\text{ PIP3. pVAV1 - k}_{\text{-22}}\text{PIP3\_pVAV1 }$$

$$\text{r}_{23}=\text{ }\frac{\text{v}_{23}.Rac\_GDP.(PIP3\_pVAV1)}{\text{k}_{23}+Rac\_GDP}\text{ }$$

$$\text{r}_{24}=\text{ }\frac{\text{v}_{24}.aSYK.PKC}{\text{k}_{24}+PKC}\text{ }$$

$$\text{r}_{25}=\text{ }\frac{\text{v}_{25}.pPKC}{\text{k}_{25}+pPKC}\text{ }$$

$$\text{ r}_{26}=\text{ k}_{26}\text{ pPKC. Grb2 - k}_{\text{-26}}\text{ pPKC\_Grb2 }$$

$$\text{ r}_{27}=\text{ k}_{27}\text{ pPKC\_Grb2. SOS - k}_{\text{-27}}\text{ pPKC\_Grb2\_SOS }$$

$$\text{ r}_{28}=\text{ k}_{28}\text{ PIP3\_pVAV1. SOS - k}_{\text{-28}}\text{ pVAV1\_PIP3\_SOS }$$

$$\text{ r}_{29}=\text{ k}_{29}\text{ RasGRP. Rac\_GTP - k}_{\text{-29}}\text{ aRasGRP }$$

$$\text{ r}_{30}=\text{ k}_{30}\text{ PIP3\_pVAV1. RasGRP - k}_{\text{-30}}\text{ PIP3\_pVAV1\_RasGRP }$$

$$\text{ r}_{31}=\text{ k}_{31}\text{ pPKC\_Grb2\_SOS. PIP3\_pVAV1\_RasGRP. Rac\_GTP - k}_{\text{-31}}\text{ pPKC\_Grb2\_SOSa. PIP3\_pVAV1 }$$

$$\text{ r}_{32}=\text{ k}_{32}\text{ PIP3\_pVAV1\_RasGRP*pVAV1\_PIP3\_SOS. Rac\_GTP - k}_{\text{-32}}\text{ pVAV1\_PIP3\_SOSa. PIP3\_pVAV1 }$$

$$\text{ r}_{33}=\text{ k}_{33}\text{ pVAV1\_PIP3\_SOS. aRasGRP - k}_{\text{-33}}\text{ pVAV1\_PIP3\_SOSa }$$

$$\text{ r}_{34}=\text{ k}_{34}\text{ pPKC\_Grb2\_SOSa - k}_{\text{-34}}\text{ pPKC\_Grb2. SOSa }$$

$$\text{ r}_{35}=\text{ k}_{35}\text{ pVAV1\_PIP3\_SOSa - k}_{\text{-35}}\text{ PIP3\_pVAV1. SOSa }$$

$$\text{ r}_{36}=\text{ k}_{36}\text{ NAP1. PIR121 - k}_{\text{-36}}\text{ NAP1\_PIR121 }$$

$$\text{ r}_{37}=\text{ k}_{37}\text{ WAVE2. Abi1 - k}_{\text{-37}}\text{ WAVE2\_Abi1}$$

$$\text{ r}_{38}=\text{ k}_{38}\text{ NAP1\_PIR121 . WAVE2\_Abi1 - k}_{\text{-38}}\text{ WAVE2\_Abi1\_NAP1\_PIR121 }$$

$$\text{ r}_{39}=\text{ k}_{39}\text{ WAVE2\_Abi1\_NAP1\_PIR121 . WRP - k}_{\text{-39}}\text{ WRP\_WAVE2\_Abi1\_NAP1\_PIR121 }$$

$$\text{r}_{40}=\text{ }\frac{\text{v}_{40}.(WRP\_WAVE2\_Abi1\_NAP1\_PIR121).Rac\_GTP}{\text{k}_{40}+Rac\_GTP}\text{ }$$

$$\text{ r}_{41}=\text{ k}_{41}\text{ EPS8. Abi1 - k}_{\text{-41}}\text{ EPS8\_Abi1 }$$

$$\text{ r}_{42}=\text{ k}_{42}\text{ EPS8\_Abi1 . PI3K - k}_{\text{-42}}\text{ EPS8\_Abi1\_PI3K }$$

$$\text{ r}_{43}=\text{ k}_{43}\text{ EPS8\_Abi1\_PI3K . SOSa - k}_{\text{-43}}\text{ EPS8\_Abi1\_SOS1\_PI3K }$$

$$\text{r}_{44}=\text{ }\frac{\text{v}_{44}.(EPS8\_Abi1\_SOS1\_PI3K)..Rac\_GDP}{\text{k}_{44}+Rac\_GDP}\text{ }$$

$$\text{r}_{45}=\text{ }\frac{\text{v}_{45}.(EPS8\_Abi1\_SOS1\_PI3K.PIP3)..Rac\_GDP}{\text{k}_{45}+Rac\_GDP}\text{ }$$

$$\text{ r}_{46}=\text{ k}_{46}\text{ WAVE2\_Abi1\_NAP1\_PIR121 . Rac\_GTP - k}_{\text{-46}}\text{ WAVE2\_Abi1\_NAP1\_PIR121\_RacGTP }$$

$$\text{ r}_{47}=\text{ k}_{47}\text{ WAVE2\_Abi1\_NAP1\_PIR121 \_RacGTP . Arp - k}_{\text{-47}}\text{ WAVE2\_Abi1\_NAP1\_PIR121\_RacGTP\_Arp }$$

$$\text{r}_{48}=\text{ }\frac{\text{v}_{48}.CDC42\_GDP..PIP2}{\text{k}_{48}+CDC42\_GDP}\text{ }$$

$$\text{r}_{49}=\text{ }\frac{\text{v}_{49}.CDC42\_GDP..PI3K}{\text{k}_{49}+CDC42\_GDP}\text{ }$$

$$\text{ r}_{50}=\text{ k}_{50}\text{ WASP. WIP - k}_{\text{-50}}\text{ WASP\_WIP }$$

$$\text{ r}_{51}=\text{ k}_{51}\text{ CDC42\_GTP. Toca - k}_{\text{-51}}\text{ CDC\_Toca }$$

$$\text{ r}_{52}=\text{ k}_{52}\text{ CDC\_Toca . WASP\_WIP - k}_{\text{-52}}\text{ CDC\_Toca\_WIP . WASP }$$

$$\text{r}_{53}=\text{ }\frac{\text{v}_{53}.WASP.aSYK\_PI3K}{\text{k}_{53}+WASP}\text{ }$$

$$\text{ r}_{54}=\text{ k}_{54}\text{ WASP. CDC42\_GTP - k}_{\text{-54}}\text{ WASP\_CDC42GTP }$$

$$\text{ r}_{55}=\text{ k}_{55}\text{ WASP\_ CDC42GTP . PIP2 - k}_{\text{-55}}\text{ WASP\_CDC42GTP\_PIP2 }$$

$$\text{ r}_{56}=\text{ k}_{56}\text{ WASP\_ CDC42GTP\_PIP2 . Arp - k}_{\text{-56}}\text{ WASP\_CDC42GTP\_PIP2\_Arp }$$

$$\text{r}_{57}=\text{ }\frac{\text{v}_{57}.PTEN\_PI3K.PIP3}{\text{k}_{57}+PTEN\_PI3K}\text{ }$$

$$\text{r}_{58}=\text{ }\frac{\text{v}_{58}.pPTEN\_PI3K}{\text{k}_{58}+pPTEN\_PI3K}\text{ }$$

$$\text{r}_{59}=\text{ }\frac{\text{v}_{59}.PTEN\_PIP2.PIP3}{\text{k}_{59}+PTEN\_PIP2}\text{ }$$

$$\text{r}_{60}=\text{ }\frac{\text{v}_{60}.pPTEN\_PIP2}{\text{k}_{60}+pPTEN\_PIP2}\text{ }$$

$$\text{r}_{61}=\text{ }\frac{\text{v}_{61}.pWASP}{\text{k}_{61}+pWASP}\text{ }$$

$$\text{ r}_{62}=\text{ }\alpha_{1}\text{-}\beta_{1}\text{. WASP-}\beta_{2}\text{. pWASP }$$

$$\text{ r}_{63}=\text{ k}_{63}\text{ WASP.\_CDC42GTP . Arp - k}_{\text{-63}}\text{ WASP\_CDC42GTP\_Arp }$$

$$\text{ r}_{64}=\text{ k}_{64}\text{ pWASP . Arp - k}_{\text{-64}}\text{ pWASP\_Arp }$$

**Rate constants**

k1=0.005 nM^-1^s^-1^; k_1=0.05 s^-1^;

k2=0.005 nM^-1^s^-1^; k_2=0.05 s^-1^;

k3=0.00005 nM^-1^s^-1^; k_3=0.00005 s^-1^;

k4=0.005 s^-1^; k_4=0.05 nM^-1^s^-1^;

k5=0.005 nM^-1^s^-1^; k_5=0.05 s^-1^;

k6=0.005 s^-1^; k_6=0.05 nM^-1^s^-1^;

k7=0.005 s^-1^; k_7=0.05 nM^-1^s^-1^;

k8=0.005 nM^-1^s^-1^; k_8=0.05 s^-1^;

v9=1.5 s^-1^; k9=500 nM;

v10=1.5 s^-1^; k10=500 nM;

v11=0.5 nM^-1^s^-1^; k11=50 nM;

v12=1.5 s^-1^; k12=500 nM;

v13=0.5 nM^-1^s^-1^; k13=50 nM;

v14=1.5 s^-1^; k14=500 nM;

k15=0.005 nM^-1^s^-1^; k_15=0.05 s^-1^;

v16=0.5 nM^-1^s^-1^; k16=50 nM;

k17=0.005 nM^-1^s^-1^; k_17=0.05 s^-1^;

k18=0.005 nM^-1^s^-1^; k_18=0.05 s^-1^;

v19=1.5 nM^-1^s^-1^; k19=500 nM;

v20=0.5 nM^-1^s^-1^; k20=50 nM;

v21=1.5 s^-1^; k21=500 nM;

k22=0.005 nM^-1^s^-1^; k_22=0.05 s^-1^;

v23=0.05 nM^-1^s^-1^; k23=50 nM;

v24=0.5 nM^-1^s^-1^; k24=50 nM;

v25=1.5 s^-1^; k25=500 nM;

k26=0.005 nM^-1^s^-1^; k_26=0.05 s^-1^;

k27=0.005 nM^-1^s^-1^; k_27=0.005 s^-1^;

k28=0.005 nM^-1^s^-1^; k_28=0.005 s^-1^;

k29=0.005 nM^-1^s^-1^; k_29=0.05 s^-1^;

k30=0.005 nM^-1^s^-1^; k_30=0.05 s^-1^;

k31=0.05 nM^-2^s^-1^; k_31=0.005 nM^-1^s^-1^;

k32=0.05 nM^-2^s^-1^; k_32=0.005 nM^-1^s^-1^;

k33=0.05 nM^-1^s^-1^; k_33=0.005 s^-1^

k34=0.5 s^-1^; k_34=0.05 nM^-1^s^-1^;

k35=0.5 s^-1^; k_35=0.05 nM^-1^s^-1^;

k36=0.005 nM^-1^s^-1^; k_36=0.05 s^-1^;

k37=0.005 nM^-1^s^-1^; k_37=0.05 s^-1^;

k38=0.005 nM^-1^s^-1^; k_38=0.05 s^-1^;

k39=0.005 nM^-1^s^-1^; k_39=0.05 s^-1^;

v40=0.5 nM^-1^s^-1^; k40=50 nM;

k41=0.005 nM^-1^s^-1^; k_41=0.05 s^-1^;

k42=0.005 nM^-1^s^-1^; k_42=0.05 s^-1^;

k43=0.05 nM^-1^s^-1^; k_43=0.005 s^-1^;

v44=0.05 nM^-1^s^-1^; k44=50 nM;

v45=0.5 nM^-2^s^-1^; k445=50 nM;

k46=0.005 nM^-1^s^-1^; k_46=0.05 s^-1^;

k47=0.005 nM^-1^s^-1^; k_47=0.05 s^-1^;

v48=0.5 nM^-1^s^-1^; k48=50 nM;

v49=0.5 nM^-1^s^-1^; k49=50 nM;

k50=0.005 nM^-1^s^-1^; k_50=0.05 s^-1^;

k51=0.005 nM^-1^s^-1^k_51=0.05 s^-1^;

k52=0.005 nM^-1^s^-1^; k_52=0.05 nM^-1^s^-1^;

v53=5 nM^-1^s^-1^; k53=50 nM;

k54=0.005 nM^-1^s^-1^; k_54=0.05 s^-1^;

k55=0.005 nM^-1^s^-1^; k_55=0.05 s^-1^;

k56=0.005 nM^-1^s^-1^; k_56=0.05 s^-1^;

v57=0.5 nM^-1^s^-1^; k57=50 nM;

v58=1.5 s^-1^; k58=500 nM;

v59=0.5 nM^-1^s^-1^; k59=50 nM;

v60=1.5 s^-1^; k60=500 nM;

v61=1.5 s^-1^; k61=500 nM;

α1=0.1 nM/s; β1=0.001 s^-1^; β2=0.002 s^-1^;

k63=0.0005 nM^-1^s^-1^; k_62=0.05 s^-1^;

k64=0.00005 nM^-1^s^-1^; k_63=0.05 s^-1^;

**Initial concentration (in nM)**

R=14.03

Src= 14.03

RS=19.67

CSK=33.7

RSC=66.3

F=100

FRSC=0

FRS= 0

PTP= 100

FRSP= 0

FRSa= 0

pP= 0

FR= 0

aSrc= 0

SYK= 100

pSYK= 0

aSYK= 0

PI3K= 23.52

aSYK_PI3K= 0

PIP2= 9.937

PIP3= 0

PTEN= 23.01

PTEN_PI3K= 54.11

PTEN_PIP2= 22.87

VAV1= 0

pVAV1= 0

PIP3_pVAV1= 0

Rac_GDP=100

Rac_GTP=0

PKC= 100

pPKC= 0

pPKC_Grb2= 0

SOS= 100

pPKC_Grb2_SOS= 0

RasGRP= 100

aRasGRP= 0

pVAV1_PIP3_SOS= 0

PIP3_pVAV1_RasGRP= 0

pPKC_Grb2_SOSa= 0

pVAV1_PIP3_SOSa= 0

NAP1=15.11

PIR121= 15.11

WAVE2= 33.28

Abi1= 1.397

WAVE2_Abi1= 4.647

NAP1_PIR121= 22.82

WAVE2_Abi1_NAP1_PIR121= 10.61

WRP= 48.53

EPS8= 68.12

EPS8_Abi1= 9.513

EPS8_Abi1_PI3K= 22.37

WRP_WAVE2_Abi1_NAP1_PIR121= 51.47

WAVE2_Abi1_NAP1_PIR121_RacGTP= 0

Arp= 40.65

WAVE2_Abi1_NAP1_PIR121_RacGTP_Arp= 0

CDC42_GDP= 0

CDC42_GTP= 1.335

WASP= 100

WIP=9

Toca= 87.29

CDC_Toca= 11.66

WASP_WIP= 89.95

CDC_Toca_WIP= 1.049

pWASP= 0

WASP_CDC42GTP= 13.35

WASP_CDC42GTP_PIP2= 13.26

WASP_CDC42GTP_PIP2_Arp=53.92

pPTEN_PI3K= 0

pPTEN_PIP2= 0

Grb2= 100

SOSa= 0

EPS8_Abi1_SOS1_PI3K=0

WASP_CDC42GTP_Arp=5.425

pWASP_Arp= 0

where,

R: integrin α2bβ3; Src: c-Src; RS: receptor-cSrc complex; CSK: CSK; RSC: receptor-cSrc-CSK complex; F: fibrinogen; FRSC: fibrinogen-receptor-cSrc-CSK complex; FRS: fibrinogen-receptor-cSrc complex; PTP: PTP-1b; FRSP: fibrinogen-receptor-cSrc-PTP1b complex; FRSa: active fibrinogen-receptor-cSrc complex; pP: phosphorylated PTP-1b; FR: fibrinogen-receptor complex; aSrc: active c-Src; SYK: SYK tyrosine kinase; pSYK: phosphorylated SYK; aSYK: active SYK; PI3K: PI3K; aSYK_PI3K: active SYK-PI3K complex; PIP2: [phosphatidylinositol 4,5-bisphosphate;](https://en.wikipedia.org/wiki/Phosphatidylinositol_4,5-bisphosphate) PIP3: [phosphatidylinositol (3,4,5)-trisphosphate;](https://en.wikipedia.org/wiki/Phosphatidylinositol_(3,4,5)-trisphosphate) PTEN: PTEN; PTEN_PI3K: PTEN-PI3K complex; PTEN_PIP2: PTEN-PIP2 complex; VAV1: Vav1; pVAV1: phosphorylated Vav1; PIP3_pVAV1: PIP3-phosphorylated Vav1 complex; Rac_GDP: RacGDP; Rac_GTP: RacGTP; PKC: protein kinase C; pPKC: phosphorylated PKC; pPKC_Grb2: phosphorylated PKC-Grb2 complex; SOS: Sosl; pPKC_Grb2_SOS: phosphorylated PKC-Grb2-Sos1 complex; RasGRP: Ras guanyl nucleotide-releasing protein; aRasGRP: active RasGRP; pVAV1_PIP3_SOS: phosphorylated Vav1-PIP3-Sos1 complex; PIP3_pVAV1_RasGRP: PIP3-phosphorylated Vav1-RasGRP complex; pPKC_Grb2_SOSa: phosphorylated PKC-Grb2-active Sos1 complex; pVAV1_PIP3_SOSa: phosphorylated Vav1-PIP3-active Sos1 complex; NAP1: NAP1; PIR121: PIR121; WAVE2: WAVE2; Abi1: Abi1; WAVE2_Abi1: WAVE2-Abi1 complex; NAP1_PIR121: NAP1-PIR121 complex; WAVE2_Abi1_NAP1_PIR121: WAVE2-Abi1-NAP1-PIR121 complex; WRP: WRP; EPS8: Eps8; EPS8_Abi1: Eps8-Abi1 complex ; EPS8_Abi1_PI3K: Eps8-Abi1-PI3K complex; WRP_WAVE2_Abi1_NAP1_PIR121: WRP-WAVE2-Abi1-NAP1-PIR121 complex; WAVE2_Abi1_NAP1_PIR121_RacGTP: WAVE2-Abi1-NAP1_OIR121-RacGTP complex; Arp: Arp2/3 complex; WAVE2_Abi1_NAP1_PIR121_RacGTP_Arp: WAVE2-Abi1-NAP1-PIR121_RacGTP-Arp2/3 complex; CDC42_GDP: Cdc42GDP; CDC42_GTP: Cdc42GTP; WASP: WASP; WIP: WIP; Toca: Toca; CDC_Toca: Cdc42GTP-Toca complex; WASP_WIP: WASP-WIP complex; CDC_Toca_WIP: Cdc42GTP-Toca-WIP complex; pWASP: phosphorylated WASP; WASP_CDC42GTP: WASP-Cdc42GTP complex; WASP_CDC42GTP_PIP2: WASP-Cdc42GTP-PIP2 complex; WASP_CDC42GTP_PIP2_Arp: WASP-Cdc42GTP-PIP2_Arp2/3 complex; pPTEN_PI3K: phosphorylated PTEN-PI3K complex; pPTEN_PIP2: phosphorylated PTEN-PIP2 complex; Grb2: Grb2; SOSa: Active Sos1; EPS8_Abi1_SOS1_PI3K: Eps8-Abi1- active Sos1-PI3K complex; WASP_CDC42GTP_Arp: WASP-Cdc42GTP-Arp2/3 complex; pWASP_Arp: phosphorylated WASP-Arp2/3 complex
